# LNX1/LNX2 proteins: functions in neuronal signalling and beyond

**DOI:** 10.1042/NS20170191

**Published:** 2018-06-07

**Authors:** Paul W. Young

**Affiliations:** School of Biochemistry and Cell Biology, Cork Neuroscience Centre, SynBioCentre, University College Cork, Cork, Ireland

**Keywords:** LNX, notch signalling pathway, neurogenesis, Numb, ubiquitin ligases

## Abstract

Ligand of NUMB Protein X1 and X2 (LNX1 and LNX2) are E3 ubiquitin ligases, named for their ability to interact with and promote the degradation of the cell fate determinant protein NUMB. On this basis they are thought to play a role in modulating NUMB/NOTCH signalling during processes such as cortical neurogenesis. However, LNX1/2 proteins can bind, via their four PDZ (PSD95, DLGA, ZO-1) domains, to an extraordinarily large number of other proteins besides NUMB. Many of these interactions suggest additional roles for LNX1/2 proteins in the nervous system in areas such as synapse formation, neurotransmission and regulating neuroglial function. Twenty years on from their initial discovery, I discuss here the putative neuronal functions of LNX1/2 proteins in light of the anxiety-related phenotype of double knockout mice lacking LNX1 and LNX2 in the central nervous system (CNS). I also review what is known about non-neuronal roles of LNX1/2 proteins, including their roles in embryonic patterning and pancreas development in zebrafish and their possible involvement in colorectal cancer (CRC), osteoclast differentiation and immune function in mammals. The emerging picture places LNX1/2 proteins as potential regulators of multiple cellular signalling processes, but in many cases the physiological significance of such roles remains only partly validated and needs to be considered in the context of the tight control of LNX1/2 protein levels *in vivo*.

## Introduction

The LNX (Ligand of NUMB Protein-X) protein family evolved from a common ancestral protein that contained a RING (Really Interesting New Gene) domain and four PDZ (PSD95, DLGA, ZO-1) domains [1]. RING domains are the most common type of catalytic domain found in E3 ubiquitin ligases [2]. These enzymes catalyse the final attachment of ubiquitin to a substrate protein during protein ubiquitination, a process that also involves an E1 (ubiquitin-activating) and an E2 (ubiquitin-conjugating) enzyme. PDZ domains mediate protein–protein interactions, usually by binding to carboxyl terminal sequence motifs of their interaction partners. The combination of RING and PDZ domains in one polypeptide is unique to LNX proteins and is reflected in the alternative name for this family: PDZRN (for PDZ and RING). Mammalian LNX1 and LNX2 were named for their ability to interact with NUMB and have retained the ancestral RING plus four PDZ domains structure [3]. LNX3, -4 and -5 (more commonly called PDZRN3, PDZRN4 and PDZK4/PDZRN4L respectively) have only one or two PDZ domains, share a unique LNX3 homology domain and are not known to interact with NUMB [1]. Thus, while LNX1/LNX2 and PDZRN3/PDZRN4/PDZK4 represent two branches of the same protein family, they are likely to have diverged significantly from each other in functional terms. The focus of this review is confined to mammalian LNX1 and LNX2 as well as the related LNX2b protein that is found in vertebrates other than eutherian mammals. Twenty years on from the cloning of the *Lnx1* gene by McGlade et al., this focus allows me to comprehensively summarize the published literature on these intriguing but incompletely understood proteins [3].

## Evolution and domain architecture of LNX1/2 proteins

**Figure 1:**
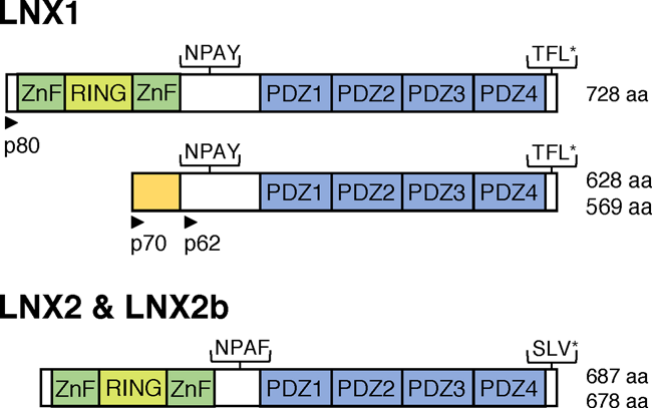
Domain architecture of vertebrate LNX1/2 proteins LNX1/2 proteins are characterized by the presence of an amino terminal RING domain flanked by two ZnF, a NUMB-binding NPAY/NPAF motif, four PDZ domains and a carboxyl terminal PDZ-binding motif (TFL* or SLV*). Several isoforms of LNX1 are expressed from two different promoters in an apparently mutually exclusive manner—LNX1p80 being expressed outside the central nervous system (CNS), while the LNX1p70 and p62 isoforms, that lack the catalytic ZnF-RING-ZnF region, are expressed primarily in neurons in the brain and spinal cord. LNX2 and LNX2b have an identical domain architecture with no known alternative isoforms. LNX2b has been lost during the evolution of eutherian mammals but is found in fish, birds and amphibians. The number of amino acids (aa) in each protein is indicated.

Vertebrate LNX1, -2 and -2b proteins have a domain architecture comprising an amino terminal RING domain flanked on each side by zinc finger motifs (ZnFs), the NUMB-binding NPAY/F motif, four PDZ domains and a carboxyl terminal PDZ binding motif [3–6] (Figure 1). A multiple sequence alignment of mouse and zebrafish LNX1, LNX2 and LNX2b proteins indicating the location of protein domains and motifs is provided as Supplementary data (Supplementary Figure S1). The poriferan *Amphimedon queenslandica* has a LNX1/2 orthologue that shares the same domain organization except for the absence of the NPAY/F motif [1]. The existence of this sponge orthologue in the most basal metazoan lineage points to the early metazoan origin of a LNX1/LNX2-like protein. The LNX1/2 PDZ domains are closely related to four carboxyl terminal domains from multiple PDZ domain containing protein-1 (MUPP1) and these proteins are likely to have shared a common ancestor. Indeed mammalian LNX1 and LNX2 interact with some of the same ligands as MUPP1, indicating possible conservation of binding specificity [1].

A *LNX1/2*-like gene is present in some, but not all, invertebrate lineages [1]. For example, species in Platyhelminthes and Mollusca have clear *LNX1/2* orthologues, whereas those in Nematoda and Arthropoda do not. This indicates that LNX1/2 proteins are not essential in all invertebrate lineages. In the vertebrate lineage, duplication of an ancestral *LNX1/2*-like gene gave rise to *LNX1, LNX2 and LNX2b*. These three genes seem to be conserved in all vertebrate lineages with the exception of the loss of *LNX2b* in eutherian mammals. This loss of *LNX2b* is quite interesting in that the *LNX2b* pseudogene contributed several exons to the non-coding *Xist* RNA that control X-chromosome inactivation in eutherian mammals [7].

The maintenance of at least two *LNX1/2*-like genes in all vertebrate lineages suggests the acquisition of essential vertebrate-specific functions. Intriguingly, vertebrate LNX1/2 proteins have either an NPAY (LNX1) or NPAF (LNX2, LNX2b) NUMB-binding motif, whereas the invertebrate proteins lack this motif [1]. One can speculate therefore that the ability to interact with and regulate NUMB represents an essential function underlying the presence of *LNX1/2* orthologues in all vertebrates. Conversely however, this same observation tells us that the ancestral LNX1/2 protein probably evolved to perform cellular function(s) not related to an interaction with NUMB. It seems likely that vertebrate LNX1/2 proteins would also have retained some of these functions. Thus an important lesson from examining the evolutionary history of LNX1/2 proteins is that, despite their name, they should not be considered a ‘one-trick pony’ and are likely to have significant functions independent of their regulation of NOTCH signalling via NUMB.

## Structural and functional analyses of LNX1/2 protein domains

### RING and zinc finger domains

The X-ray crystallographic structure of the LNX2 RING domain was determined by Nayak and Sivaraman in 2015 [4] and provides a number of interesting mechanistic insights into the ubiquitin ligase function of the LNX1/2 family. Conserved cysteine and histidine residues are present in the sequences immediately before and after the RING domain. Initially, it had been speculated that the RING domain and the sequence immediately C-terminal to it might constitute an example of a novel phosphotyrosine binding Hyb domain, similar to that found in the E3 ubituitin ligase Hakai [8]. However, the subsequent crystal structure revealed the presence of a ZnF on either side of the RING domain, but did not indicate the presence of an Hyb domain [4]. Instead, the ZnF C-terminal to the RING domain was noted to most closely resemble a similarly arranged domain in the E3 ubiquitin ligase TRAF6. Interestingly, the ZnF N-terminal to the RING domain has an unusual open circle configuration that lacks any secondary structure. The presence of this type of ZnF N-terminal to a RING domain seems to be unique to LNX1, LNX2 and LNX2b proteins and appears to be present in invertebrate LNX1/2 orthologues, including sponges (based on the conservation of the zinc co-ordinating Cysteine and Histidine residues). This motif is not conserved in PDZRN3/LNX3 or PDZRN4/LNX4, although these proteins do appear to contain a ZnF to the C-terminal side of their RING domains. Mutational analysis of LNX2 showed that this unusual amino terminal ZnF was essential for its E3 ubiquitin ligase activity, whereas the carboxyl terminal ZnF was not [4]. Together these data indicate that the open circle ZnF amino-terminal to the RING domain is characteristic of the LNX1/LNX2 proteins and of functional importance.

Using LNX2 autoubiquitination as a readout of E3 ubiquitin ligase activity Nayak and Sivaraman [4] saw that UbcH5b was a preferred E2 ubiquitin conjugating enzyme for LNX2 (as had been shown for LNX1 [9–11]). Interestingly, the formation of all seven forms of ubiquitin chain linkages could be catalysed by LNX2/UbcH5b in *in vitro* ubiquitination assays. They also showed for the first time that LNX2 could indeed ubiquitinate NUMB, something that had previously only been shown for LNX1. LNX2 autoubiquitination diminished but did not abolish this activity. Another finding from their study was that the ZnF-RING-ZnF LNX2 construct was present as a dimer in both the crystal structure and in solution. However, dimerization was not required for LNX2 autoubiquitination activity.

Having determined the structure of the LNX2 RING domain with its flanking ZnF motifs, Nayak and Sivaraman [5] have recently taken a further step towards elucidating the detailed catalytic mechanism of LNX1/2 proteins by solving the structure of the LNX1 ZnF-RING-ZnF region in complex with ubiquitin-loaded Ubc13. Ubc13 was shown to act as an E2 ubiquitin conjugating enzyme for LNX1 that promoted the formation of K63-linked polyubiquitin chains—the first demonstration of LNX1/2 proteins utilizing an E2 ubiquitin conjugating enzyme other than UbcH5b. The structure of LNX1 in a complex with Ubc13 conjugated to ubiquitin revealed that both ZnF motifs in LNX1 are involved in forming contacts with Ubc13 and both are required for LNX1 catalytic activity. This contrasts with LNX2 in which the amino but not the carboxyl terminal ZnF is essential for activity [4]. Like LNX2, the ZnF-RING-ZnF region of LNX1 is dimeric and the structure reveals how dimerization of LNX1 stabilizes a closed conformation of the E2 ubiquitin conjugating enzyme and ubiquitin, which is thought to enhance ubiquitin transfer to substrates. Together these two studies by Nayak and Sivaraman [4,5] have highlighted unique features of the catalytic mechanisms of LNX1/2 proteins. Based on these findings, future studies of LNX1/2-mediated ubiquitination in cellular contexts will need to carefully consider the type of ubiquitin chain linkages that are attached to a given LNX1/2 substrate, the particular E2 ubiquitin conjugating enzyme that is involved and the roles of autoubiquitination and dimerization in regulating ubiquitin ligase activity and substrate recognition.

### PDZ domains

Apart from the RING domain, structures of a number of individual PDZ domains are available in the Protein Data Bank (PDB; http://www.rcsb.org). Specifically, structures of mouse LNX1 PDZ2 (PDB ID: 3VQf, 3VQG), human LNX1 PDZ3 in complex with a peptide ligand from the coxsackievirus and adenovirus receptor (CAR) (PDB ID: 3B76) and human LNX2 PDZ2 (PDB ID: 2VWR, 5E1Y, 5E11, 5E21, 5E22) have been submitted to the PDB. Most of these structures are not associated with any journal publication, though interestingly LNX2 PDZ2 was used in a recent, highly innovative study in which the application of strong electric field pulses to protein crystals was combined with time-resolved X-ray crystallography in order to probe the internal protein mechanics of a PDZ domain [12]. The motions induced within the PDZ domain by the electric-field were postulated to reflect the conformational changes associated with ligand binding. Further analysis of these motions may provide insights into the mechanism of ligand binding by this PDZ domain and through comparison with the LNX1 PDZ2 structure may elucidate the structural basis for the differential binding of certain ligands to PDZ2 from LNX1 compared with LNX2 [13,14].

A number of studies have examined the general binding specificities and potential intra- and intermolecular interactions of LNX1/2 proteins via their PDZ domains. These studies are described in this section, whereas individual PDZ-mediated interactions with other proteins are discussed later. Gao et al. have examined the consensus binding properties of mammalian LNX1 and LNX2 PDZ domains through yeast two-hybrid screening of random carboxyl terminal peptide libraries, which then allowed them to identify and verify candidate interacting proteins [9,15]. The binding specificities of PDZ domains to carboxyl terminal sequences can be categorized into four classes based on the last four amino acids of the binding peptides [16]. LNX1 PDZ1 fits the consensus for Class I PDZ domains, -x-[Ser/Thr]-x-Φ* (where x is any amino acid and Φ is a hydrophobic residue) [9]. Interestingly, both LNX1 and LNX2 have carboxyl terminal motifs (-Gly-Thr-Phe-Leu* and -Gly-Ser-Leu-Val* respectively) that fits this consensus (Figure 1). Indeed, the carboxyl terminal 33 amino acids of LNX2 can bind to LNX1 and LNX2 [6] and for both LNX1 [9] and LNX2 [6], this carboxyl terminal interaction was mapped to PDZ1. LNX1 PDZ1 can also interact with constructs encoding the PDZ4 and the carboxyl terminus of both LNX1 and LNX2, though whether this interaction is mediated by binding of PDZ1 to LNX1/2 carboxyl terminal sequences or via a PDZ–PDZ interaction has not been determined definitively [9]. In fact, a subsequent study found that LNX2 PDZ1 could bind to internal sequence motifs [17] and LNX1 PDZ1 is thought to bind an internal sequence in the NUMB PTB (phosphotyrosine binding) domain [10]. Thus, the question of whether interactions of LNX1 and LNX2 PDZ1 domains do indeed involve the carboxyl terminus of other proteins needs to be carefully examined. In any case, regardless of the precise mode of binding, the aforementioned LNX–LNX interactions could: (i) allow LNX1 and LNX2 to form either homo- and heterodimers or (ii) result in the carboxyl terminal end of an individual LNX1/2 protein folding back to interact with its first PDZ domain, depending on whether they occur inter- or intramolecularly.

In contrast with PDZ1, LNX1 PDZ2 and PDZ3 as well as LNX2 PDZ2 are able to bind peptides belonging to multiple classes of PDZ binding motifs (Class I and II ± class III) [9]. This broad specificity may explain why the majority of LNX1/2 ligands identified in the literature to date bind to these PDZ domains. No clear consensus binding specificity for carboxyl terminal peptide sequences could be determined for LNX2 PDZ1 and PDZ3, and no peptides binding to PDZ4 from either LNX1 or LNX2 were identified [9]. Thus only for PDZ2 were consensus binding sequences determined for both LNX1 and LNX2. Interestingly, while the binding specificity of PDZ2 from the two mammalian LNX1/2 proteins overlaps, it is not identical. The consensus sequence is -x—x-Ψ-[Val/Ile/Leu/Cys]* for LNX1 PDZ2 compared with -Φ—[Val/Ile/Leu/Ser/Thr]-[Val/Ile/Leu]-[Val/Ile/Leu]* for LNX2 PDZ2 (where Ψ is an aromatic residue). This suggests the possibility of differential binding of LNX1 and LNX2 to certain ligands. Indeed this was subsequently shown in proteomics studies in which the preferential binding of LNX1 PDZ2 to ligands with carboxyl terminal Cysteine residues in particular was noted [13,14,18]. Such LNX1- or LNX2-specific interactions may underlie specialized functions of LNX1/2 paralogues in vertebrates.

The fact that yeast two-hybrid screens failed to identify any peptides that bind to PDZ4 from either LNX1 or LNX2 [9] is noteworthy, and to our knowledge there is only one report in the literature of proteins binding to LNX1/2 PDZ4 domains [18]. The paucity of PDZ4 interacting proteins may be explained by the finding that this PDZ domain, in LNX1 at least, can bind to phospholipids [18]. This binding was specific for phosphatidylinositol phosphate lipids over other common membrane lipids. Thus LNX1 PDZ4, and perhaps LNX2 PDZ4, may interact primarily with lipids rather than proteins and could potentially play a role in localizing LNX1/2 proteins to specific membrane regions in cells, though this remains speculative at present.

While we have learned a lot from the study of individual LNX1/2 domains, a structure of a full-length LNX1/2 protein would now be extremely informative. Such a structure would resolve the nature of PDZ1-mediated intra- and/or intermolecular interactions of LNX1/2 proteins. It would hopefully also reveal how the 3D arrangement of PDZ domains in a full-length LNX1/2 protein might orient ligands of specific PDZ domains as potentials substrates for ubiquitination by the ZnF-RING-ZnF domain.

## *LNX1/2* mRNA and protein expression

### *LNX1/2* mRNA expression

In this section, I focus on mammalian *LNX1* and *LNX2*, the expression of *lnx2b* in zebrafish is discussed separately below. Northern blot analysis revealed expression of murine *Lnx1* mRNA in adult heart, brain, lung, skeletal muscle and kidney with little or no message detected in spleen, liver and testis [3]. Human *LNX1* mRNA was likewise detected in adult heart, brain and kidney as well as the placenta and pancreas, whereas much lower levels were detected in lung, liver and skeletal muscle [19]. Thus *Lnx1* mRNA seems to be expressed in many adult tissues. Notably, both these and subsequent studies [20] showed that different *Lnx1* transcripts are expressed in the brain and spinal cord compared with other tissues. These transcripts are predicted to give rise to 70 and 62 kDa proteins that lack the ZnF-RING-ZnF domain present in the 80 kDa protein that is expressed in other tissues (Figure 1 and Supplementary Figure S1). Rice et al. [6] showed that *Lnx2* mRNA is also widely expressed in adult mouse tissues and examined embryonic expression of both *Lnx1 and Lnx2* mRNAs by *in situ* hybridization. At E11.5 *Lnx2* but not *Lnx1* was expressed in the neuroepithelium of the developing forebrain. In E14.5 embryos *Lnx2* is strongly expressed in the forebrain and in many other regions of the developing embryo while *Lnx1* expression is confined to the brain and spinal cord at this stage. Both *Lnx1* and *Lnx2* mRNA expression persists in the brain post natally with *Lnx2* expression particularly prominent in the cerebellum [6]. *Lnx1* and *Lnx2* expression in the murine brain has been shown to be predominantly neuronal, though some expression in oligodendrocytes was observed in the cerebellum [20]. Overall these studies reveal both *Lnx1* and *Lnx2* mRNAs to be quite widely expressed postnatally, with the earliest embryonic expression of both genes being in the CNS. This early expression in the embryonic brain and spinal cord hints at potential functions in neural development, possibly through regulation of NUMB proteins.

A number of studies have reported differential expression of *Lnx1* mRNAs in particular situations, providing potential hints regarding LNX1 function. For example *Lnx1* mRNA is relatively abundant in the pineal gland compared with other tissues and shows alterations in day time compared with night time expression levels [21,22]. No analysis of LNX1 function in the pineal gland has been reported however. Interestingly, several reports describe high relative expression of *Lnx1* in diverse stem/progenitor cell populations including quiescent muscle satellite cells [23], oligodendrocyte precursor cells [24] and limbal stem cells of the eye [25]. However, LNX1 protein levels were not examined in these studies and thus the functional significance of these observations remains unclear.

Another largely unexplored area of question is how the transcription of *Lnx1 and Lnx2* genes is regulated. Alternative promoters give rise to strict differential expression of the LNX1p70 and p62 encoding transcripts in neurons compared with LNX1p80 encoding mRNA*s* in other tissues, but the transcription factors acting at either promoter have not been described. Kohn et al. [26] reported correlated expression of *LNX1* with components of tight junctions across human cancer cell lines, suggesting a shared epithelial function for LNX1 with this set of tight junction proteins. Possible mechanisms co-ordinating the expression of this set of proteins were not described however. The transcription factor RUNX2 plays a role in controlling *Lnx2* expression in osteoblasts [27] and the transcriptional repressor GLI3 may suppress *Lnx2* expression during neuronal differentiation based on the increased *Lnx2* levels observed in *Gli3*^−/−^ mice [28]. Overall though our understanding of the transcriptional regulation of mammalian *LNX1/2* genes is very incomplete.

### LNX1/2 protein expression

Despite the widespread expression of *LNX1/2* mRNAs, LNX1/2 proteins appear to be present *in vivo* at relatively low levels. Detection of endogenous LNX1/2 proteins by Western blotting generally requires immunoprecipitation from tissue lysates [13,20,29–33], though LNX2 can be detected directly in brain lysates from mice at embryonic and early postnatal but not adult stages [13,28]. Both LNX1 and LNX2 can be detected directly by Western blotting in colorectal cancer (CRC) cell lines [34,35] but their detection in other cell lines has not been widely reported. Immunostaining for endogenous LNX1 protein has been reported in the perisynaptic Schwann cells of neuromuscular junctions [33], while LNX2 protein was detected broadly in most organs of embryonic day 16.5 mice [30]. LNX1 and LNX2 were both detected by immunostaining in the acrosome of murine spermatozoa [36].

Immunoprecipitation from murine brain lysates confirmed the expression of LNX1p62 and p70 proteins, albeit at low levels [20]. This may be partly explained by the presence of upstream ORFs and other sequence elements that suppress protein translation within the 5′-UTRs of the mRNAs encoding these isoforms [20]. Upstream ORFs in mRNAs can divert ribosomes away from translation of the main coding sequence [37]. Such mechanisms do not appear to regulate translation of LNX1p80 (or LNX2), but LNX1p80 protein levels may be influenced by rapid protein turnover via proteasomal degradation [20]. Together these mechanisms may explain, at least in part, the low levels of LNX1 proteins detected *in vivo*. The postnatal down-regulation of LNX2 protein levels in the brain [13] despite the continued expression of *LNX2* mRNA [6], suggests that either high rates of protein turnover or other mechanisms operating at the level of protein translation may control adult LNX2 protein levels. Possible regulatory mechanisms could include the repression of translation by non-coding RNAs and one report has identified a miRNA, *miR-939*, that may target *Lnx2* message [38].

The exclusive expression in the CNS of LNX1 protein isoforms (p62 and p70) that lack the catalytic ZnF-RING-ZnF region is intriguing. The p70 isoform is found across diverse vertebrate species and the short amino terminal sequence unique to this isoform is highly conserved [1]. Since they lack catalytic activity but retain the NPAY motif and PDZ protein interaction domains, these neuronally expressed isoforms might be expected to have opposite effects from LNX1p80 or LNX2 towards substrate proteins—interacting with but not ubiquitinating them, and perhaps protecting them from ubiquitination by LNX2 in situations in which they are co-expressed. However, in some cases it appears that LNX1p70, despite lacking a catalytic domain, can nevertheless promote the ubiquitination of certain interacting proteins (LIPRIN-α1 and KLHL11), most likely by acting as a scaffold to recruit other ubiquitin ligases [14]. In addition, the neuronal LNX1 isoforms may have scaffold functions that are not related to ubiquitination. For example, LNX1p70 was able to facilitate the endocytosis of junctional adhesion molecule (JAM) 4 (JAM4), possibly by mediating the formation of a tripartite complex with NUMB [39]. Similarly, a role for LNX1p80 in sensitizing HK-2 cells to TGF-β induced epithelial to mesenchyme transition (EMT) was shown to be independent of its ubiquitin ligase activity. While these examples are not in a neuronal context, they highlight the potential for ubiquitination independent functions of the neuronal LNX1 p70 and p62 isoforms.

Overall the low expression levels of LNX1 and LNX2 proteins, and the possible lack of correlation between *LNX1/2* mRNA and protein levels *in vivo* have significant implications for interpreting reports on the role of LNX1/2 proteins in disease, and the physiological relevance of the many interactions of LNX1/2 proteins that have been identified to date. The seemingly tight control of LNX1/2 protein levels suggest that LNX1/2 proteins (i) may have general functions in the many cell types, but that they are only required at extremely low levels or (ii) have very specific roles in certain cell types in which they are expressed at higher levels but in a temporally or spatially restricted manner. The fact that LNX1/2 proteins are normally present *in vivo* at relatively low levels must be kept in mind when interpreting experiments that rely heavily on LNX1/2 overexpression.

## Interaction of LNX1/2 proteins with NUMB and NUMB-like

### LNX1/2 proteins as ligands of NUMB

LNX1 was first identified in a yeast two-hybrid screen to identify proteins that bind to the PTB domain of murine NUMB [3]. The interaction was mapped to the sequence NPAY within LNX1 which matches a consensus binding sequence for other PTB domains. However, phosphorylation of the tyrosine residue was not required for NUMB binding since mutation of the tyrosine in this motif to phenylalanine did not affect the interaction. It was also shown that the PTB domain of NUMB-like, a closely related paralogue of NUMB, could also bind LNX1. When LNX2 was subsequently cloned it was also shown to bind both NUMB and NUMB-like via its NPAF motif [6], again highlighting the lack of a requirement for tyrosine phosphorylation. The interaction of LNX1/2 NPAY/F motifs with NUMB seems to be quite specific since the binding of other PTB domains to LNX1/2 proteins via this motif has not been reported. The only exception to this is that the PTB domain from SHC was shown to bind to a LNX1 peptide, but only when the tyrosine in the NPAY motif was phosphorylated [3]. Whether such phosphorylation of LNX1 occurs *in vivo* is not known, so the physiological relevance of a LNX1–SHC interaction is unclear.

The ubiquitin ligase activity of LNX1 was confirmed and NUMB was shown to be a substrate for LNX1-mediated ubiquitination [11]. Ubiquitination of NUMB by LNX2 was subsequently shown for both the mammalian and zebrafish proteins [4,32]. Both LNX1 and LNX2-mediated ubiquitination appear to target NUMB for proteasomal degradation [11,32]. In the case of LNX1 it was found that the first PDZ domain was required for ubiquitination of NUMB [11]. Four major mammalian NUMB isoforms (p65, p66, p71 and p72) exist. While all NUMB isoforms can bind to LNX1, only the p66 and p72 isoforms that contain an 11-amino acid insert in their PTB domain are ubiquitinated by LNX1 [10]. This region was shown to interact with the PDZ1 region of LNX1 and this interaction, in addition to the canonical binding of the PTB to the LNX1 NPAY motif, seems to be required for ubiquitination of NUMB p66 and p72 by LNX1. The four NUMB isoforms exhibit developmental and tissue specific expression patterns [40]. Given the isoform specificity of NUMB ubiquitination, it is important when examining LNX1 function to know which isoform(s) of NUMB are expressed in the cell or tissue type of interest. NUMB-like lacks the PTB insertion found in NUMB p66 and p72 suggesting perhaps that it may not be a LNX1 substrate, despite having been shown to interact with LNX1 [3]. At present it is not known if LNX2 exhibits that same isoform specificity for NUMB ubiquitination as LNX1. These are important questions regarding the interactions of LNX1/2 proteins with NUMB and NUMB-like that need to be addressed.

### LNX1/2 proteins as regulators of NOTCH signalling via NUMB

NUMB interacts with the NOTCH receptor and can negatively regulate NOTCH signalling in several contexts. NUMB is thought to achieve this inhibition of NOTCH signalling by modulating either the endocytosis of NOTCH or the trafficking of NOTCH post-endocytosis, however the exact mechanism is a matter of debate [41]. Given NUMB’s role in antagonizing NOTCH signalling, the ubiquitination of NUMB by LNX1/2 proteins and its subsequent proteolytic degradation would be expected to promote NOTCH signalling. Indeed, it was shown that LNX1 overexpression caused a 30% increase in NOTCH signalling as measured using a luciferase reporter assay based on the Hes-1 NOTCH target gene [11]. This enhancement of NOTCH signalling correlated with NUMB ubiquitination and degradation. However, one caveat with this experiment is that LNX1 was overexpressed to a degree that may exceed physiological levels.

Importantly, some evidence that a loss of LNX1/2 function can lead to altered NOTCH signalling has emerged more recently. In zebrafish embryos expressing a dominant negative form of LNX2 it was found that NUMB levels were increased and the number of cells exhibiting NOTCH signalling was decreased, as assessed using a transgenic fluorescent protein-based reporter [32]. In a different study, LNX2 knockdown in bone-derived macrophages resulted in an increase in NUMB protein levels, a decrease in NOTCH2 levels and decreased expression of the NOTCH target gene *Hes1* [42]. However, some other studies have not seen alterations in NOTCH signalling as a consequence of altered LNX1/2 levels. In Gli3 knockout-mice LNX2 up-regulation is associated with a dramatic loss of NUMB, but the authors comment that expression of the NOTCH target gene *Hes5* is unaffected and rather point to other functions of NUMB to explain the phenotypes observed in these mice [28]. In another report, LNX2 knockdown in a pancreatic cancer cell line did not affect NOTCH signalling, but rather altered the MAPK/ERK signalling activity [43].

These observations make it clear that relating increased or decreased LNX1/2 protein expression to alterations in NOTCH signalling in a given context is not straightforward. Firstly, LNX1/2 may or may not affect NUMB levels depending on the particular isoform(s) of NUMB present; secondly, redundancy of function between LNX1/2 isoforms and between NUMB and NUMB-like need to be considered and thirdly, even if NUMB levels are altered this could affect pathways other than NOTCH signalling. Finally, it is possible that LNX1/2 may modulate NUMB and/or NUMB-like function independently of triggering their degradation, for example by altering NUMB or NUMB-like subcellular localization. Nevertheless, LNX1/2 proteins certainly can act as activators of NOTCH signalling through the ubiquitination of NUMB, but this mechanism must be carefully examined in each cellular context for the reasons outlined above.

### Roles of LNX1/2 proteins in neurogenesis and neuronal differentiation

NUMB functions as a cell fate determinant in many types of neural progenitor cells, as well as in non-neural cells. During neurogenesis asymmetric localization of NUMB in dividing neural progenitors causes it to partition predominantly into one of the daughter cells—specifying a distinct fate for this cell by antagonizing NOTCH signalling [44]. For example, one daughter cell may differentiate while the other retains its progenitor status. A number of studies that have highlighted potential roles for LNX1/2 proteins in regulating neurogenesis and neuronal differentiation are summarized in this section.

While examining knockout mice lacking the Sonic hedgehog signalling component GLI3, Wang et al. [28] uncovered a potential role for LNX2 in neurogenesis in the subventricular zone (SVZ)—a neurogenic region in both embryonic and adult mammals. In the SVZ ependymal cells, as well as neural stem cells, originate from radial glial cells, but in Gli3^−/−^ mice defects in the specification of ependymal cells were observed. These mice exhibit dramatically decreased levels of NUMB protein, which may at least in part explain the phenotype observed. Intriguingly, Gli3^−/−^ mice have increased LNX2 levels, particularly in the SVZ, leading the authors to speculate that LNX2 overexpression in Gli3^−/−^ mice may target NUMB for proteolytic degradation. While this mechanism seems plausible, a causal relationship between LNX2 and NUMB levels was not definitively demonstrated. Nevertheless, this is an interesting finding that begs the question of whether LNX2 plays a role in normal SVZ development beyond this proposed role in Gli3^−/−^ animals.

The interaction of contactin-associated protein-4 (CASPR4) with LNX2 has also been proposed to play a role in neuronal differentiation [45]. CASPR4 is a transmembrane protein that is a member of the neurexin protein superfamily. It is expressed in the developing cortex and neural progenitor cells in the SVZ. LNX2 was found to bind to the cytoplasmic domain of CASPR4 and both proteins were found to inhibit proliferation and promote the differentiation of cultured neural progenitor cells based on siRNA and overexpression experiments. Co-expression of CASPR4 and LNX2 in the SVZ was shown by immunostaining. LNX2 expression could rescue a deficit in neuronal differentiation caused by CASPR4 knockdown, leading the authors to hypothesize that LNX2 functions downstream of CASPR4 in promoting neuronal differentiation. The mechanism beyond this was not explored however, and so it remains to be seen whether or not it involves LNX2 modulation of NUMB or NOTCH signalling.

A distinct role for LNX1/2 proteins in cortical neurogenesis has been proposed based on their interaction with SHOOTIN1 [46]. SHOOTIN1 localizes to the apical surface of the ventricular zone in dividing radial glial cells that serve as progenitors for cortical neurons. SHOOTIN1 overexpression biases dividing cells towards symmetrical divisions that generate two progenitor cells, whereas SHOOTIN1 knockdown led to cell divisions that give rise to more neurons. In addition, SHOOTIN1 knockout mice exhibit significant defects in cortical development. NUMB is known to control the balance between the maintenance of progenitor pools and neuronal differentiation. Therefore the regulation of NUMB and NOTCH signalling by LNX1/2 proteins was investigated as a potential mechanism to explain these findings. LNX1 and LNX2 proteins co-localized with SHOOTIN1 in the ventricular zone and could be immunoprecipitated in a complex with NUMB and SHOOTIN1 from brain lysates. Significantly, SHOOTIN1 enhanced the ability of LNX1 to ubiquitinate NUMB and enhanced NOTCH signalling when overexpressed in embryonic brains, while SHOOTIN1 knockout mice have increased NUMB levels and decreased NOTCH signalling as assessed by a NOTCH reporter assay. Together these data lead to a model in which SHOOTIN1, by enhancing the ubiquitin ligase activity of LNX1/2 proteins, promotes the degradation of NUMB and thereby enhances NOTCH signalling.

The examples above indicate several potential roles for LNX1/2 proteins in regulating neurogenesis and neuronal differentiation and fit with the well-established role of NUMB in such processes. However, none could be considered definitive proof of such a role *in vivo* during normal brain development. Notably, gross brain morphology was found to be normal and no changes in NUMB levels were observed in mice lacking both LNX1 and LNX2 in the nervous system [13]. Furthermore, ependymal cell differentiation appeared normal in the SVZ of these mice. These observations suggest that the roles played by LNX1/2 proteins in neurogenesis and neuronal differentiation must be relatively subtle or may be compensated for in some way in the absence of LNX1/2 proteins. More detailed analysis of LNX1/LNX2 DKO mice, guided by the findings of the above studies, should further elucidate such roles.

### A role for LNX2 in osteoclast differentiation

A possible role for mammalian LNX1/2 proteins in the differentiation of osteoclasts that seems to involve the modulation of NOTCH signalling via NUMB has recently been described [42]. This investigation stemmed from the observation that both *Lnx1* and *Lnx2* mRNAs are significantly up-regulated during osteoclast differentiation *in vitro*. To probe the requirement for LNX2 the authors employed lentiviral transduction of shRNAs to knockdown LNX2 expression in bone marrow derived macrophages. This drastically decreased osteoclast formation *in vitro* and increased apoptosis of pre-osteoclasts. LNX2 knockdown also dampened the activation of the signalling pathways that promote osteoclast formation, namely M-CSF induced ARK and AKT activation, as well as RANKL induced NF-κB and JNK activity. Notably LNX2 knockdown caused an increase in NUMB levels and decreased levels of the NOTCH2 intracellular domain, as well as decreased expression of the NOTCH target gene *Hes1*. NOTCH signalling is known to play multiple roles in maintaining bone homoeostasis and controlling osteoclast differentiation [47,48]. These findings suggest that the effects of LNX2 on osteoclast formation could be mediated by modulation of NOTCH2 signalling through NUMB. It will be extremely interesting to see if these findings in an *in vitro* differentiation system can be confirmed *in vivo*, perhaps by examining osteoclast differentiation and homoeostasis in detail in *Lnx2* knockout mice.

## Interactions of LNX1/2 proteins with ligands other than NUMB and NUMB-like

**Figure 2:**
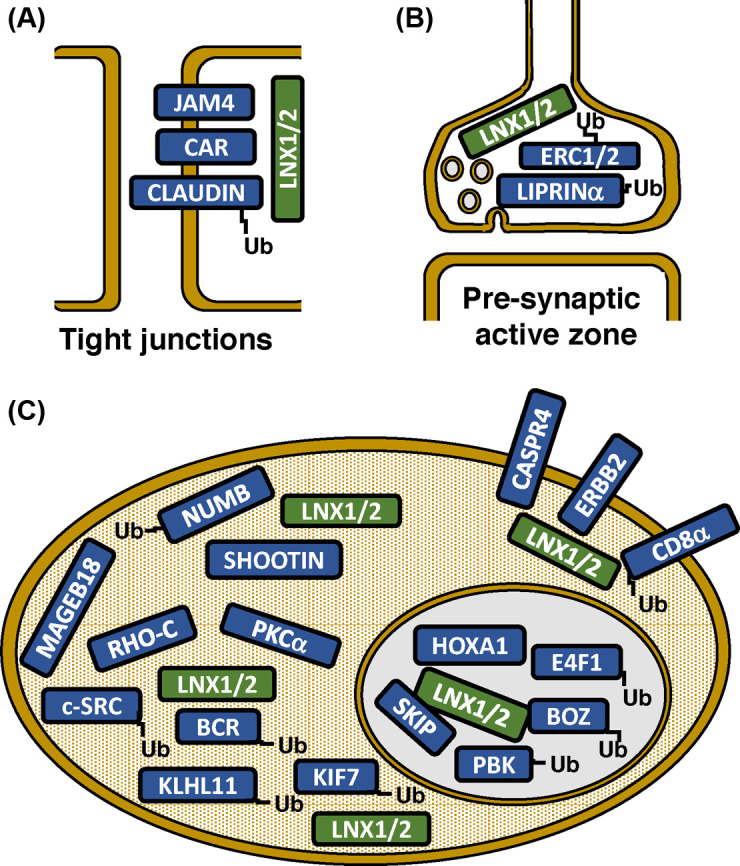
Interactions of LNX1/2 proteins A selection of some of the better characterized LNX1/2 interacting proteins are shown grouped according their subcellular localization. (**A**) Tight junctions of epithelial cells; (**B**) The presynaptic cytomatrix of the active zone (CAZ) complex in neurons; (**C**) Interactions of LNX1/2 proteins with cytoplasmic and plasma membrane signalling molecules as well as with nuclear proteins involved in transcriptional regulation. Only one or a few of the depicted interactions are likely to occur in any one cell type at a given time. ‘Ub’ indicates interacting proteins that are known to be substrates for ubiquitination by LNX1/2 proteins. See the main text and Table 1 for further details and references.

Several hundred proteins have been described as potentially interacting with LNX1/2 proteins and the overwhelming majority of these interactions are mediated by the LNX1/2 PDZ domains [9,13,18,20]. Some of these putative interactions were identified in high throughput screens using the yeast two-hybrid system or chemically synthesized peptides [9,15,18,49,50]. Clustering analysis for LNX1 interacting proteins based on gene ontology annotations have provided some insights into potentially novel LNX1 functions [9,18]. More recently affinity purification combined with MS has been employed to characterize the LNX1 interactome in the HEK293 cell line and LNX1 and LNX2 PDZ2 interacting proteins from mouse brain lysates [13,14]. Given the inherent limitations of these high throughput approaches I focus below on those interactions that have at least been verified using full-length proteins expressed in mammalian cells (Table 1). Furthermore, I have attempted to group these interactions according to their cellular context (Figure 2).

**Table 1:** Known interactions of LNX1/2 proteins

Interacting protein	Description/function	Binds to:	Domain(s) involved	Methods used	Substrate for ubiquitination	References
**NUMB**	Cell fate determinant	LNX1, LNX2	NPAY/F, PDZ1	Y2H, GST-PD, Co-IP(h)	Yes; proteasomal degradation	[3,4,6,10,11]
**NUMB-like**	Cell fate determinant	LNX1, LNX2	NPAY/F motif	Y2H, GST-PD, Co-IP(h)	n/d	[3,6]
**SHOOTIN**	Polarity protein	LNX1, LNX2	PDZ2,3,4	Protein array, Co-IP(h,e)	n/d	[46]
**CASPR4**	Neurexin family protein	LNX2	PDZ2	Y2H, GST-PD, Co-IP(h), Co-L	n/d	[45]
**CLAUDIN-1, 2, 17**	Tight junction	LNX1	PDZ1, PDZ2	Y2H, GST-PD, Co-IP(h)	Yes; endocytosis	[9,51]
**JAM4**	Tight junction	LNX1	PDZ2	Y2H, Co-IP(h), Co-local	n/d	[39]
**CAR**	Tight junction	LNX1, LNX2	PDZ2	Y2H, GST-PD, Co-IP(h)	n/d	[30,36,52]
**ERC1, ERC2**	Presynaptic	LNX1, LNX2	PDZ2	Y2H, Co-IP(h), GFP-PD, Co-L	Yes	[13,14,56]
**LIPRIN-α1, -α3**	Presynaptic	LNX1, not -2	PDZ2	Co-IP(h) GFP-PD	Yes; not degraded	[13,14]
**ERBB2**	Receptor Tyrosine kinase	LNX1	PDZ1-4	Y2H, Co-IP (h,e)	n/d	[33]
**CD8-α**	T-cell co-receptor	LNX1, LNX2	PDZ1/2	Y2H, Co-IP(e), GST-PD, Co-L	Yes; lysosomal degradation	[29]
**KCNA4**	K^+^ channel	LNX1, LNX2	PDZ1	Y2H, Co-IP(h)	n/d	[15,18]
**EPHA7**	Receptor Tyrosine kinase	LNX1, LNX2	PDZ1-4	Y2H, GFP-PD	n/d	[1,14,50]
**Bozozok**	Transcription factor	LNX2b	n/d	Co-IP(h)	Yes; degradation	[69]
**E4F1**	Transcription factor	LNX1, -2, -2b	n/d	Co-IP(h), GST-PD	Yes; not degraded	[71]
**TCF3**	Transcription factor	LNX2b	n/d	Co-IP(h)	n/d	[71]
**TLE3**	Transcription factor	LNX2b	n/d	Co-IP(h)	n/d	[71]
**HDAC1**	Histone deactylase	LNX2b	n/d	Co-IP(h)	n/d	[71]
**NP9**	Nuclear protein	LNX1	PDZ2-4	Y2H, GST-PD, Co-L	n/d	[58]
**SKIP**	Nuclear protein	LNX1	n/d	Y2H, Co-IP(h), Co-L	n/d	[61]
**HOXA1**	Transcription factor	LNX2	n/d	Y2H, Co-IP(h), BiFC	n/d	[63]
**KLHL11**	Kelch-like	LNX1, LNX2	PDZ2	GFP-PD	Yes	[14]
**KIF7**	Kinesin	LNX1, not -2	PDZ1-4	GFP-PD	Yes	[14]
**SYNGAP1**	GTPase activating protein	LNX1, LNX2	PDZ1, PDZ2	Y2H, GFP-PD	n/d	[1,14]
**SRGAP2**	GTPase activating protein	LNX1, LNX2	PDZ2	GFP-PD	Yes (weak)	[13,14]
**MID2**	Ubiquitin ligase	LNX1, not -2	PDZ1-4	GFP-PD	n/d	[14]
**AKAP13**	Scaffold protein	LNX1, not -2	PDZ2	GFP-PD	n/d	[14]
**PBK**	MAP kinase kinase	LNX1, LNX2	PDZ1 or PDZ1-4	Y2H, Co-IP(h)	Yes; proteasomal degradation	[9,15,18]
**BCR**	GTPase activating protein	LNX1	PDZ3	Y2H	Yes, proteasomal degradation	[9]
**c-SRC**	Tyrosine kinase	LNX1	PDZ3, PDZ1	PDZ array, Co-IP(h,e), Co-L	Yes, proteasomal degradation	[31]
**RHO-C**	GTPase	LNX1	PDZ1	Y2H, Co-IP(h), Co-L	n/d	[59]
**PAK6**	Ser/Thr kinase	LNX, LNX2	PDZ2,4	Y2H, Co-IP(h)	n/d	[15,18]
**PLEKHG5**	RhoGEF protein	LNX1, LNX2	PDZ1,3	ProtoArray, Co-IP(h)	n/d	[18]
**PKCα**	Ser/Thr kinase	LNX1, LNX2	PDZ2,4	Peptide Array, Co-IP(h)	n/d	[18,50]
**TYK2**	Non-receptor Tyrosine kinase	LNX1, LNX2	PDZ2	Y2H, Co-IP(h)	n/d	[15,18]
**RNase-L**	Endoribonuclease	LNX1	PDZ1-4	Y2H, Co-IP(h)	No	[84]
**MAGEB18**	Tumour antigen	LNX1	-	TAP, Co-IP(h)	n/d	[66]

Only interactions that have been verified using full-length proteins expressed in mammalian cells are listed. For interacting proteins that are LNX1/2 substrates, the consequence of ubiquitination is stated (if known). Abbreviations: BCR, breakpoint cluster region protein; Co-IP(h) or Co-IP(e), co-immunoprecipitation of heterologously expressed or endogenous proteins respectively; Co-L, co-localization in cells or tissues; GFP-PD or GST-PD, ‘pull down’ experiment using a GFP or GST tag respectively; n/d, not determined; PBK, PDZ-binding kinase; SKIP, Ski interacting protein; Y2H, yeast two-hybrid.

### Interactions of LNX1/2 proteins with components of cell–cell junctions

The cytoplasmic domains of several components of epithelial tight junctions including CLAUDINS [15,51], junctional adhesion molecules (JAMs) [39] and CXADR/CAR [52] have been shown to bind to LNX1/2 PDZ domains and LNX1 has been implicated in regulating E-cadherin expression [53]. These studies are discussed below.

CLAUDINs comprise a large family of transmembrane proteins involved in the formation of tight junctions that maintain epithelial and endothelial barrier functions. CLAUDIN-1 cytoplasmic domain was found to interact with LNX1 via its carboxyl terminal PDZ binding motif [51]. LNX1 overexpression in epithelial MDCK cells caused a dramatic removal of CLAUDINs from tight junctions, whereas other tight junction components were not affected. LNX1 overexpression also caused defects in tight junction morphology and adversely affected epithelial barrier function. LNX1 was able to ubiquitinate CLAUDIN-1, -2 and -4 and LNX1-mediated ubiquitination of CLAUDIN-1 in MDCK cells was increased in the presence of the lysosome inhibitor chloroquine. This indicates that CLAUDIN redistribution upon LNX1 overexpression is a consequence of ubiquitination triggered endocytosis and subsequent lysosomal degradation. Overall this study highlights the potential of LNX1 to potently regulate CLAUDINs at tight junctions, although a significant caveat is that this conclusion is based largely on LNX1 overexpression and so the extent of this regulation at physiological levels of LNX1/2 expression needs to be examined.

JAM4 is a member of the immunoglobulin superfamily of adhesion molecules that localizes to tight junctions. JAM4 was shown to interact via its carboxyl terminal PDZ binding motif with PDZ2 of LNX1 and LNX1 was seen to partly co-localize with JAM4 and other tight junction components in epithelial cells [39]. LNX1p70, which lacks the catalytic RING domain, was shown to facilitate the endocytosis of JAM4 and this effect was dependent on the presence of NUMB. Furthermore, LNX1, NUMB and JAM4 were shown to form a tripartite complex, leading to a model whereby LNX1 acts as a scaffold to link NUMB and JAM4, thereby promoting endocytosis of JAM4 independent of LNX1-mediated ubiquitination of NUMB.

CAR like JAM4 is a member of the immunoglobulin superfamily that can interact with LNX1 via its cytoplasmic tail [52]. CAR is named based on it being a receptor for both the coxsackievirus and adenovirus but its normal function appears, at least in part, to relate to maintaining tight junction integrity where it may act as a homophilic adhesion molecule [54,55]. The cytoplasmic tail of CAR was shown to bind to both LNX1 and LNX2 via PDZ2 [30,52]. When co-expressed with CAR LNX1 was recruited to cell–cell junctions where it co-localized with CAR. *In vivo* both CAR and LNX2 proteins are present in several tissues in embryonic day 16.5 mice and in particular co-expression of both protein was noted in a subset of blood vessels [30]. CAR and LNX1/2 proteins were both detected in spermatozoa from murine testis where LNX2 but not LNX1 co-localized with CAR in the acrosome [36]. The demonstration of co-localization of endogenous LNX2 protein with CAR *in vivo* is a major strength of these studies but the functional consequence of the interaction of LNX1/2 proteins with CAR either in epithelial or sperm cells remains to be established. Notably mice lacking LNX2 appear to have normal fertility, suggesting that LNX2 is not absolutely required for sperm development and fertilization in any case [13]. Similarly, a LNX2 null mutation did not affect reproductive function in zebrafish [32].

These studies suggest multiple ways in which LNX1/2 proteins might be involved in the formation or remodelling of tight junctions in epithelial cells. This may be relevant in the context of EMTs that occur developmentally and that also contribute to tumour development and metastasis [39]. Interestingly LNX1 was shown to make kidney proximal tubular epithelial cells more sensitive to TGF-β induced EMT, an effect that was independent of E3 ubiquitin ligase activity [53]. LNX1 expression enhanced the TGF-β induced transcriptional down-regulation of the epithelial marker E-cadherin and up-regulation of vimentin. Furthermore, LNX1 expressing cells showed increased migration rates. These findings illustrate another way in which LNX1/2 proteins may modulate epithelial cell function.

### Interaction of LNX1/2 proteins with ERC/CAST and LIPRIN-α in the presynaptic active zone complex

Potential functions of LNX1/2 proteins in presynaptic nerve terminals are suggested by the characterization of interactions of LNX1/2 proteins with several presynaptic proteins. The finding that both LNX1 and LNX2 are predominantly expressed in neurons [20] suggests that interactions of LNX1/2 proteins with presynaptic components, may be physiologically relevant. These interactions include ERC/CAST and LIPRIN-α proteins—two components of the presynaptic cytomatrix of the active zone (CAZ) complex that functions in synapse maturation and the regulation of synaptic vesicle release [13,14,56]. ERC2 (CAST1) was shown to bind to LNX1-PDZ2 via a carboxyl terminal PDZ-binding sequence and transfected LNX1 co-localized with endogenous ERC2 in nerve terminals of cultured hippocampal neurons [56]. When co-expressed in heterologous cells ERC2 recruits LNX1 into punctate structures and LNX1 was purified in a Triton X-100 insoluble fraction from which it is excluded when expressed alone [14,56]. LNX2 also binds to ERC2 and both LNX1 and LNX2 can bind ERC1 [13]. LNX1p80 can ubiquitinate ERC2 [14], though LNX2 may be more likely to play this role at synapses since LNX1p80 is not expressed in the CNS.

Proteomic analyses of the LNX1 interactome revealed an interaction of LNX1 with LIPRIN-α proteins [13,14]. This interaction was not shared by LNX2 and was dependent on the carboxyl terminal –YSC motif of LIPRIN-α1. LIPRIN-α1 is a substrate for ubiquitination by LNX1p80 and surprisingly the neuronally expressed LNX1p70, that lacks the catalytic RING domain, is able to promote LIPRIN-α1 ubiquitination, possibly by recruiting another E3 ubiquitin ligase [14]. In any case, ubiquitination of LIPRIN-α1 by LNX1 in HEK293 cells did not significantly alter LIPRIN-α1 protein levels, suggesting that LNX1 does not target LIPRIN-α1 for either proteasomal or lysosomal degradation in this context at least. Thus while both the ERCs and LIPRIN-αs are substrates for ubiquitination by LNX1/2 proteins, the functional consequences of this remain to be elucidated.

A number of other proteins that play important roles in synapse formation and function have also been reported to interact with LNX1/2 proteins. These include MYCBP2 (PAM/HIGHWIRE/RPM-1), a well-known regulator of synapse formation [14] and CASPR2 which regulates the maturation and function of GABAergic and dopaminergic synapses [57], though its interaction with LNX1/2 has been characterized in the context of neurogenesis [45]. Proteins that regulate presynaptic functions would be good candidates to play a role in the anxiety-related phenotype observed in LNX1/LNX2 DKO mice (see below) [13].

### Interaction of LNX1 with ERBB2 in perisynaptic Schwann cells

ERBB2 is a member of the epidermal growth factor receptor family of receptor tyrosine kinases with important signalling functions in many contexts during embryonic development, including the differentiation of myelinating Schwann cells in the peripheral nervous system. ERBB2 was found to interact with the PDZ domain region of LNX1 and endogenous ERBB2 could be co-immunoprecipitated with LNX1 from mouse brain lysate [33]. Immunostaining for LNX1 in the peripheral nervous system showed a striking localization of LNX1 in perisynaptic Schwann cells at neuromuscular junctions, with little or no staining in myelinating Schwann cells along motor axons. Signalling from the axon-derived ERBB2 ligand neuregulin promotes the differentiation of myelinating Schwann cells. The specific localization of LNX1 in persisynaptic Schwann cells suggests that it may play a role in modulating neuregulin/ERBB2 signalling to establish and or maintain their non-myelinating status. It appears that it is the RING domain-containing LNX1p80 isoform that is present in perisynaptic Schwann cells [20], however, whether LNX1 can ubiquitinate ERBB2 has not been reported. Nevertheless, the very restricted localization of LNX1 protein is intriguing, as very few perisynaptic Schwann cell specific markers are known.

### Interaction of LNX1/2 proteins with CD8 in T cells

CD8 is a T-cell co-receptor that can stabilize the interaction of the T-cell receptor with the class I MHC on antigen presenting cells and recruit effector proteins to the T-cell receptor. The cytoplasmic tail of CD8-α was found to bind to both LNX1 and LNX2 [29]. The interaction required the CD8-α carboxyl terminal amino acids and the interaction was mapped to the LNX1/2 PDZ domains. Co-immunoprecipitation of the endogenous proteins was possible from the HPB-ALL T-cell line. Both LNX1 and LNX2 promoted ubiquitination of CD8-α and overexpression of either LNX1 or LNX2 protein was able to reduce the plasma membrane levels of transfected CD8-α. LNX1/2 proteins and CD8-α co-localized with markers of both endosomes and lysosomes in these experiments and levels of CD8-α were reduced in a manner that could be partly reversed using a lysosomal inhibitor. Taken together these results suggest that, at least when overexpressed, LNX1 and LNX2 can cause ubiquitination of CD8-α, thereby triggering its endocytosis and targeting for lysosomal degradation. While *Lnx1* and *Lnx2* mRNA was detected in purified T cells, the levels of LNX1 or LNX2 proteins were not assessed and are likely to be significantly lower than are achieved by heterologous expression. It will be important therefore to show that the endogenous levels of LNX1/2 proteins in T cells can affect CD8-α levels and /or subcellular localization, perhaps by examining T cells under conditions of LNX1/2 knockdown or loss of function.

### Interaction of LNX1/2 proteins with nuclear proteins and transcriptional regulators

The best characterized interactions of LNX1/2 proteins with transcriptional regulators are those of zebrafish LNX2b (described separately). However, a few interactions of mammalian LNX1 and LNX2 with nuclear proteins have been described. Several reports describe LNX1 as being partly localized in the nucleus [31,58,59], and LNX1 has been implicated in cell cycle control and modulation of transcription [53,59,60]. These observations are suggestive of functions for LNX1 in the nucleus.

Ski interacting protein (SKIP/SNW1) was identified in a yeast two-hybrid screen using PDZ1 of LNX1 as a bait [61]. Employing exogenous expression of both proteins the interaction was confirmed by co-immunoprecipitation and both proteins were detected in the nucleus. SKIP/SNW1 functions as a transcriptional regulator that can modulate transcription associated with several signalling pathways. While the SKIP–LNX1 interaction was not characterized further it is interesting to note that SKIP, through interaction with the NOTCH intracellular domain, plays a role in the assembly of NOTCH transcriptional activation complexes [62]. This suggests a possible mechanism whereby LNX1 might regulate NOTCH signalling independently of NUMB.

Armbruester et al. [58] described an interaction of LNX1 with the nuclear protein NP9 from the human endogenous retrovirus K. NP9 is a 74-amino acid protein that is predominantly expressed in tumours but the functional relevance of its interaction with LNX1 remains to be determined. An interaction of mouse HOXA1 transcription factor with LNX2 has also been described [63]. This association was found by yeast two-hybrid screening and confirmed by co-immunoprecipitation and bimolecular fluorescence complementation in cultured cells, but as for NP9, the functional significance of the interaction is unknown.

PDZ-binding kinase (PBK/TOPK) is another nuclear protein that has been shown to interact with LNX1 [9,15,18]. PBK functions as a mitogen-activated protein kinase kinase that can down-regulate the tumour suppressor protein p53 and is up-regulated in many human cancers [64,65]. PBK could be co-immunoprecipitated with LNX1 employing exogenous expression of both proteins [18]. In a separate study, PBK was shown to be ubiquitinated by LNX1 which caused its degradation in a proteasome-dependent manner [9]. Knockdown of LNX1 expression in HEK293T cells using siRNA led to elevation of PBK levels and enhanced cell growth. Conversely, LNX1 overexpression decreased PBK levels and reduced cell growth. Furthermore, LNX1 knockdown sensitized cells to doxorubicin-induced apoptosis, while LNX1 overexpression protected them [9]. Together these findings suggest that ubiquitination by LNX1 can moderate the growth promoting and anti-apoptotic effects of PBK by targeting it for proteasomal degradation.

### Interaction of LNX1/2 proteins with components of intracellular signalling pathways

The non-receptor tyrosine kinase c-SRC can interact via its carboxyl terminus with both PDZ1 and PDZ3 of LNX1 [31]. The two proteins co-localized at cell–cell contacts, membrane ruffles and cytoplasmic punctae when co-expressed in HEK293 cells. LNX1 was able to enhance c-SRC ubiquitination and this was increased further in the presence of a proteasome inhibitor. Furthermore, LNX1 expression caused a decrease in the levels a constitutively active form of c-SRC. c-SRC is able phosphorylate both full-length LNX1 and an amino terminal LNX1 fragment lacking the PDZ domains, though the functional significance of LNX1 phosphorylation is not clear. Thus c-SRC and LNX1 may mutually regulate each other with LNX1 potentially targeting activated c-SRC for proteasomal degradation.

Zheng et al. [59]. found an interaction of LNX1-PDZ1 with RHO-C—a member of the small GTPase superfamily that plays key roles in regulating the actin cytoskeleton. They found that LNX1/2 recruited RHO-C to the nucleus and that RHO-C could reverse LNX1-mediated enhancement of AP-1 transcriptional activity, though the mechanisms underlying these effects were not investigated. Guo et al. [9] described breakpoint cluster region protein (BCR) as a substrate for ubiquitination by LNX1. BCR is a RHOGAP (RHO GTPase activating protein) for the small GTPases RAC1 and CDC42. LNX1 expression caused a reduction in BCR protein levels that could be reversed by a proteasomal inhibitor, suggesting that ubiquitination by LNX1 targets BCR for degradation. Wolting et al. describe several other cytoplasmic signalling proteins that bind to LNX1 including the serine/threonine kinases PAK6 and PKCα, the RHOGEF (RHO guanine nucleotide exchange factor) PLEKHG5 and the non-receptor tyrosine kinase TYK2 [18]. An interaction of LNX1 with the tumour antigen protein MAGE-B18 has also been described and the interaction site on LNX1 was mapped to a region between the ZnF/RING domains and the first PDZ domain [66]. These interactions were not characterized further however. Finally, a proteomic analysis identified the A-kinase anchoring protein AKAP13 and members of the TRIM family of E3 ubiquitin ligase (MID2/TRIM1 and TRIM27) in the LNX1 interactome [14].

It clear from the examples above that LNX1 has the ability to interact with, and in some cases ubiquitinate, key components of intracellular signalling pathways. Going forward, it will be interesting to determine in which *in vivo* cellular contexts these interactions and potential modulatory influences of LNX1/2 proteins on cell signalling are relevant.

## *In vivo* functional analysis of LNX1/2 proteins

### Mammalian LNX1 and LNX2

The first LNX1/2 loss-of-function mouse model was an isoform-specific LNX1 knockout generated by Lexicon Pharmaceuticals as part of a large-scale gene knockout programme. In this mouse deletion of *LNX1 exon3* eliminates expression of the neuronally expressed LNX1p70 and p62 proteins, but is not expected to affect LNX1p80 expression in other tissues [13]. This mouse was subjected to comprehensive phenotyping which did not reveal any significant findings apart from an increased percentage of B1-like B cells in peritoneal lavage (https://www.mmrrc.org/catalog/sds.php?mmrrc_id=32436). However, only basic neurological and behavioural tests were performed, so subtle neural defects cannot be ruled out. The sole immunological abnormality reported is difficult to reconcile with the fact that only the neuronal LNX1 isoforms are eliminated in these mice.

A *LNX2* knockout mouse was subsequently generated in my laboratory [13,20]. Since this mouse did not exhibit any obvious phenotype it was crossed to the *LNX1*^exon3−/−^ line mentioned above to generate LNX1/LNX2 double knockout (DKO) mice in which LNX2 is eliminated globally in all tissues, while the loss of LNX1 is expected to be restricted to the exon 3-containing LNX1 p70 and p62 isoforms in the CNS. These mice thus represent a loss-of-function model to study neuronal functions of mammalian LNX1 and LNX2. LNX1/LNX2 DKO mice are viable, healthy and fertile and do not exhibit gross neuroanatomical defects [13]. Cortical organization and lamination appear normal, suggesting that the overall processes of neurogenesis and neuronal differentiation are not significantly perturbed. Levels of NUMB protein in brain lysates were unaltered and the development of the neurogenic SVZ including ependymal cell differentiation appeared normal. This indicates that while LNX2 up-regulation in Gli3^−/−^ mice was associated with decreased NUMB levels and abnormalities in SVZ formation [28] (see above), LNX2 expression during normal development does not appear to affect NUMB function in SVZ formation. In addition, no defects were observed in cellular organization in the cerebellum of LNX1/LNX2 DKO mice, a region with prominent *Lnx1 and Lnx2* mRNA expression.

When subjected to behavioural testing LNX1/LNX2 DKO mice showed normal learning, motor and sensory function [13]. Motor co-ordination and motor skill learning was intact as assessed by the rotarod test, despite the fact that *Lnx1* and *Lnx2* mRNAs are expressed in the motor cortex, spinal cord and cerebellum—areas that are involved in motor function [6,20]. The major finding of the behavioural analysis was that LNX1/LNX2 DKO mice exhibited decreased anxiety-related behaviour in certain behavioural paradigms (open field and elevated plus maze) but not others (light-dark box) [13]. These tests can be regarded as measuring overlapping, but partially distinct, aspects of anxiety-related behaviour. Thus, the reduced anxiety-like behaviour observed for DKO mice seems to be a very specific phenotype that is only observed in a subset of anxiety-related testing paradigms. At present the molecular mechanisms and the neural circuitry defect that may underlie this phenotype remain unclear. Although NUMB levels in total brain lysate seem unaffected, the loss of LNX1/2 proteins might alter NUMB levels in a spatially or temporally restricted manner. Alternatively, other aspects of NUMB function such as its subcellular localization might be perturbed without affecting total NUMB levels. However, given that LNX1/LNX2 DKO mice lack obvious NUMB-related abnormalities, one needs to consider the possibility that their reduced anxiety-related behaviour could be a consequence of the loss of interactions of LNX1 and/or LNX2 with proteins other than NUMB. Candidates to play such a role include interacting proteins with known synaptic or neuronal signalling functions such as the presynaptic active zone constituents ERC1, ERC2 and LIPRIN-αs, as well as FCHSD2 (nervous wreck homologue), SRGAP2, SYNGAP1 and EPHA7 (see Table 1). Going forward, the molecular mechanisms and the brain regions underlying this unexpected function for LNX1/2 proteins in the regulation of anxiety-related behaviour will hopefully be elucidated.

The only other significant phenotype observed in these LNX1/2 DKO was that they weighed approximately 10% less than wildtype animals by adulthood [13]. This weight difference became apparent around weaning age. While this weight difference could be caused by a neural abnormality affecting appetite or exercise for example, it could also be a consequence of the loss of LNX2 outside the CNS in these mice. LNX2 plays a role in pancreas development in zebrafish (see below) [32]. Abnormal pancreatic function could conceivably affect weight gain, though a role for LNX2 in the gut or in regulating some aspect of metabolism could also be responsible. Interestingly a genome-wide association study (GWAS) linked *LNX2* to weight gain in cattle [67] and another study identified *LNX2* as a growth-related quantificative trait locus in the Chinese mitten crab [68]. A closer analysis of organ function outside the CNS may clarify the basis for the decreased weight observed in in LNX1/2 DKO mice.

### Zebrafish LNX2 and LNX2b

The role of Lnx2b (Lnx-like) in zebrafish development have been extensively examined by Ro and Dawid (2009, 2010, 2011) [69–71], making Lnx2b the best understood member of the LNX1/2 family in terms of its physiological functions. Using *lnx2b* antisense morpholinos, it was initially observed that depletion of Lnx2b in early stage zebrafish embryos lead to excessive dorsalization, as assessed by examination of several dorsoventral markers [69]. Lnx2b was shown to interact Bozozok (BOZ), a homoeobox domain containing protein that promotes embryonic dorsalization and is important for anterior–posterior neural patterning. Lnx2b mediated ubiquitination of BOZ was shown to trigger its degradation via the proteasome. Lnx2b overexpression caused embryonic defects including anterior notochord and forebrain malformations reminiscent of *bozozok* loss-of-function mutations and could counteract the dorsalization promoted by BOZ overexpression. Furthermore, when BOZ overexpression was combined with Lnx2b depletion ectopic expression of *goosecoid*, a key regulator of dorsal–ventral patterning, was observed in the presumptive ectoderm [70]. Taken together these data support a model whereby maternally derived Lnx2b antagonizes BOZ function during gastrulation in zebrafish embryonic development by targeting it for proteasomal degradation. As a consequence of this moderating effect on BOZ, Lnx2b prevents the expansion of dorsal organizer gene expression into ectodermal tissues and thus plays an essential role in dorso–ventral patterning.

A distinct role for Lnx2b in modulating the expression of the caudal homeobox gene *cdx4* during zebrafish embryogenesis has also been shown [71]. Cdx/caudal transcription factors, including Cdx4, function in the patterning of caudal embryonic structures. *Cdx4* expression is repressed by Tcf3 in the absence of Wnt signalling, whereas the transcriptional regulator E4f1 was shown to de-repress *cdx4*. Lnx2b can interact with E4f1, but promotes repression of *cdx4* —counteracting the action of E4f1. While Lnx2b can ubiquitinate E4f1 in cell-based ubiquitination assays, this did not lead to E4f1 degradation and its E3 ubiquitin ligase activity was not required to antagonize the de-repression of *cdx4* by E4f1. Thus the interaction of Lnx2b with E4f1 appears to be sufficient to modulate the Tcf3 repression complex on the *cdx4* promoter, although interactions of Lnx2b with other components of the complex could play a role.

A third function for Lnx2 and -2b proteins during zebrafish development has also been revealed by Dawid et al. [32]. In this study, a redundant role for Lnx2b and Lnx2 (Lnx2a) in the differentiation of exocrine cells in the pancreas was identified. Embryos in which expression of both Lnx2 and Lnx2b was disrupted or those expressing a putative dominant-negative form of Lnx2 lacking the RING domain, had a marked loss of pancreatic exocrine cells as assessed by expression of the *Ptf1a* transcription factor that is required for differentiation of these cells. Other exocrine markers such as trypsin were also drastically diminished, whereas endocrine cell differentiation seemed unaffected. The specificity of this phenotype tallied with the expression of *lnx2* mRNA in the ventral pancreatic bud from which exocrine cells are derived, but not in the dorsal bud from which endocrine cells arise. The mechanism underlying this phenotype appears to involve LNX2/2b-mediated regulation of Notch signalling via Numb, based on the observations that Numb knockdown substantially rescued the phenotype and that increased levels of Numb and decreased Notch signalling were seen in pancreatic cells of embryos expressing dominant negative Lnx2. Destabilization of Numb by Lnx2 and Lnx2b is proposed to promote Notch signalling in ventral bud-derived cells which appears to be important in the specification and/or proliferation of the exocrine precursor cell population [32].

Together these studies in the zebrafish model represent the best mechanistic descriptions of LNX1/2 protein function *in vivo.* A key question is whether these functions are shared in other lineages, particularly in eutherian mammals that lack LNX2b. The absence of a clear mammalian *Bozozok* orthologue suggests that this function of Lnx proteins in very early embryonic patterning may be specific to fish. By contrast, Ro and Dawid [71] demonstrated that human LNX1 and LNX2 can interact with and ubiquitinate human E4F1, suggesting a potentially broader role for the LNX1/2 family in modulating E4F1 function beyond zebrafish. This and the question of whether LNX2 and/or LNX1 play a role in modulating NOTCH signalling during mammalian pancreas development are thus important topics for future research.

## Disease associations of LNX1/2 proteins

### Potential roles for LNX1 in brain tumours, cell cycle control and malignant transformation

A number of reports have highlighted altered LNX1 expression in the context of brain tumours, although in most cases a causative role for LNX1 in driving tumorigenesis has not been demonstrated. Down-regulation of *LNX1* mRNA was noted in gliomas compared with healthy brain tissue [71], though this might reflect the fact that *LNX1* and *LNX2* mRNA are expressed predominantly in neurons [20] and as such would be present at lower levels in gliomas that are derived from glia. By contrast, other reports demonstrate amplifications of a chromosomal region (4q12) containing the *LNX1* gene in brain tumours and osteosarcoma xenografts [72–74]. It is noteworthy however that this region contains a number of potentially cancer promoting receptor tyrosine kinase encoding genes (c-*KIT, PDGFR-α* and *VEGFR2*) and so amplification of *LNX1* may be incidental. A few cases with specific amplification of *LNX1* were reported [73] and some *LNX1* missense mutations were found [72], however overexpression of LNX1 at the mRNA or protein level was not shown in these studies. Zheng et al. (2011) [60] highlighted a potential role for LNX1 as a cell cycle regulator. Knockdown of *LNX1* mRNA expression in HEK cells using siRNA led to cell cycle arrest and caused alterations in the expression levels of several cancer associated genes. Nie et al. [53] reported that overexpression of LNX1 could promote TGF-β induced EMT in cultured epithelial cells, an activity that did not require the LNX1 E3 ubiquitin ligase activity. This represents another potential mechanism by which LNX1 might promote tumour formation. However in the absence of data demonstrating increased expression of *LNX1* mRNA and particularly LNX1 protein in tumours, the question of whether LNX1 actually promotes a cancer phenotype *in vivo* remains unclear and further evidence is required to demonstrate a compelling causative role for LNX1 in brain tumours.

#### Involvement of LNX1 and LNX2 in CRC

The most comprehensive studies linking LNX1/2 proteins to tumorigenesis are in relation to CRC [34,35]. The human *LNX2* gene maps to chromosome 13q12.2—a region that is frequently amplified in CRC. Examining genes that are amplified in this chromosomal region, Camps et al. [34] found *LNX2* mRNA to be overexpressed in CRC compared with normal colon mucosa as well as in CRC cell lines. Furthermore, it was shown that *LNX2* knockdown using siRNA reduced the viability of CRC cell lines and induced apoptosis. Using genome-wide expression profiling they observed dysregulation of 680 genes upon silencing of *LNX2* in a CRC cell line (SW480) that exhibits chromosome 13 amplification. The expression of multiple target genes of both the NOTCH and WNT/β-catenin signalling pathways were prominently decreased and alterations of upstream components of these pathways were noted at both the mRNA and protein levels. In particular, TCF7L2, a transcription factor that is a major effector of WNT signalling, and many of its target genes were down-regulated in *LNX2* knockdown cells. Similarly, levels of NOTCH and the NOTCH ligand JAG1 as well as several NOTCH target genes were significantly decreased. Thus, LNX2 has the ability to simultaneously regulate two signalling pathways known to play critical roles in CRC tumorigenesis. Surprisingly, decreased NUMB protein levels were observed upon LNX2 knockdown—the opposite of what one might expect for NUMB as a substrate for LNX2-mediated ubiquitination and an inhibitor of NOTCH signalling. Thus, the observed changes in NOTCH signalling cannot be explained simply by LNX2 modulating NUMB levels. Nevertheless, the findings suggest a credible model whereby overexpression of LNX2 as a consequence of *LNX2* gene amplification promotes NOTCH and WNT/β-catenin signalling, which drives cell proliferation and prevents apoptosis in CRC cells [34].

A more recent study has now also implicated LNX1 in CRC [35]. Here the authors observed that *LNX1* mRNA and protein were down-regulated in a population of putative cancer stem cells (side population cells) isolated from the CRC cell line HT29. Cancer stem cells in tumours are proposed to give rise to cells with highly proliferative and invasive phenotypes. *LNX1* knockdown increased the number of cancer stem cells and led to the formation of more colonospheres in extreme limited dilution assays, and more tumours in mice injected with *LNX1* knockdown HT29 cells. This suggested that LNX1 is a negative regulator of cancer stemness in CRC. Seeking a mechanism for this effect the authors examined LNX1 interacting proteins and found that knockdown of CAR, a known LNX1 ligand [52], caused a decrease in cancer stem cells in the same system. *LNX1* knockdown leads to increased CAR protein levels, suggesting that CAR may be involved in mediating the effect of LNX1 on cancer stemness, although the mechanism by which LNX1 regulated CAR levels and whether it involved LNX1-mediated ubiquitination of CAR was not examined. Finally, this study showed that tamoxifen increased *LNX1* gene expression and could inhibit colonosphere formation in HT29 cells. Overall these findings suggest that LNX1 may act as a tumour suppressor and that up-regulating LNX1 expression may represent a novel therapeutic strategy to negatively regulate cancer stemness in CRC.

Interestingly, a previous study of cancer stem cells in glioblastoma reported *LNX1* mRNA to be up-regulated in CD133− compared with CD133+ stem cells, suggesting that an involvement of LNX1 in cancer stemness may not be specific to CRC [75]. Furthermore, a GWAS identified a single nucleotide polymorphism (SNP) within the *LNX2* promoter region that was linked to increased susceptibility to diffuse large B-cell lymphoma [76] and another GWAS linked a SNP in the *LNX2* gene to differential responses to the chemotherapeutic drug cytosine arabinoside in lymphoblastoid cells [43]. These studies hint at a possible role for LNX2 in haematological tumours.

#### Association of LNX1/2 with immune function and infectious disease

A couple of studies have found associations of LNX1 and LNX2 with infectious diseases. A GWAS found intronic SNPs in *LNX1* that are associated with susceptibility to Kawasaki disease—an inflammatory paediatric condition that causes damage to the corony arteries and is thought to be triggered by an unidentified infection [77]. This study also noted differences in whole blood *LNX1* transcript levels in acute compared with convalescent Kawasaki disease. A different report found that blood levels of both *LNX1* and *LNX2 mRNAs* were significantly elevated in chronic compared with acute Q fever [78]. Q fever is caused by *Coxiella burnetti* infection and the acute form usually resolves by itself, whereas chronic Q fever develops as an endocarditis and is associated with an impaired immune response. The authors suggest that *LNX1/2* expression could be used as a prognostic biomarker in Q fever patients. These reports, along with the previously discussed interaction of LNX1/2 proteins with CD8 [29] suggest that LNX1/2 proteins may modulate immune response in certain contexts.

#### Miscellaneous disease associations of LNX1/2 genes

Differential expression of *LNX2* mRNA expression in placental tissue was found to be associated with preeclampsia in pregnancy [79], while an epigenome-wide association study identified a CpG site within the *LNX2* gene to be hypomethylated in smokers [80]. The functional significance of these findings remains to be established.

## Conclusion

Two decades of research have highlighted a multitude of potential functions for LNX1/2 proteins, based largely on their ability to interact with many binding partners (Figure 2 and Table 1). However, the physiological relevance of most of these putative functions are yet to be validated *in vivo* (with the notable exception of studies of zebrafish Lnx1/2 proteins). While our understanding of some basic aspects of LNX1/2 function is still incomplete, it is improving. It is now appreciated that LNX1/2 protein levels are tightly regulated. We are beginning to understand the differential functions of LNX1, LNX2 and LNX2b, as well as the neuronal and non-neuronal LNX1 isoforms. Fantastic structural insights into the mechanisms of LNX1/2 ubiquitin ligase activity have emerged in the past few years. The LNX1/2 interactome has been thoroughly explored using a variety of complementary approaches. Loss-of-function analyses of mammalian LNX1/2 proteins in both cellular and mouse models have been published. Many pieces of the puzzle are falling into place.

Going forward armed with this knowledge and the new research tools that are available, researchers in this field can be optimistic about putting more pieces of this puzzle together. The currently available and newly generated LNX1 and LNX2 knockout mice can be used to explore the significance of putative functions of LNX1/2 proteins *in vivo.* The molecular basis for the subtle phenotypes already observed in mice lacking LNX1 and LNX2 proteins in the CNS can be dissected more precisely. The generation of mice lacking both LNX1 and LNX2 in all tissues will clarify proposed roles for LNX1/2 proteins in immune function and bone homeostasis and allow the role for LNX1/2 proteins in pancreas differentiation to be explored in mammals. Further structural studies will hopefully reveal structures of full-length LNX1/2 proteins and/or structures of LNX: substrate complexes. Translational studies will be also be needed to tease out the disease associations of LNX1/2 proteins, particularly in CRC, where both LNX1 and LNX2 have been implicated. These studies will determine whether LNX1/2 proteins are worth considering as potential drug targets, particularly as methods to inhibit both E3 ubiquitin ligases and PDZ domain interactions with small molecules develop [81–83].

## Supplementary Material

Supplemental Figure 1

## Abbreviations

BCR: breakpoint cluster region protein
BOZ: Bozozok
CAR: coxsackievirus and adenovirus receptor
CASPR4: contactin-associated protein-4
CNS: central nervous system
CRC: colorectal cancer
DKO: double knockout
EMT: epithelial to mesenchyme transition
GWAS: genome-wide association study
JAM: junctional adhesion molecule
JNK: c-Jun N-terminal kinase
LNX: ligand of NUMB protein X
M-CSF: macrophage colony stimulating factor
MDCK: Madin-Darby Canine Kidney cells
MUPP1: multiple PDZ domain containing protein-1
NF-κB: nuclear factor kappa B
PBK: PDZ-binding kinase
PDB: Protein Data Bank
PDZ: PSD95, DLGA, ZO-1
PDZRN: PDZ and RING
PTB: phosphotyrosine binding
RANKL: receptor activator of nuclear factor kappa B ligand
RING: really interesting new gene
SKIP: Ski interacting protein
SHC: Src homology 2 domain-containing
SNP: single nucleotide polymorphism
SVZ: subventricular zone
TGF-β: fransforming growth factor β
ZnF: zinc finger motif
