# Supplementary material for: LNX1/LNX2 proteins: functions in neuronal signalling and beyond
Source: Neuronal Signal. 2018 Jun 7;2(2):NS20170191. doi: 10.1042/NS20170191 (PMC7373230; doi:10.1042/NS20170191)
Supplement: Supplemental Figure 1 [file ns-02-ns20170191_supp.pdf]

Supplemental Figure 1. Multiple sequence alignment of zebrafish and mouse LNX1/2 proteins.

The positions of the RING and PDZ domains as defined by SMART (http://smart.embl-heidelberg.de) are indicated below the alignment, as are the location of the zinc finger (ZnF) motifs either side of the RING domain. Conserved residues involved in co-ordination of zinc ions in the X-ray crystal structure of human LNX2 are indicated by an asterisk (Protein Data Base ID: 5DIN; Nayak and Sivaraman (2015) Oncotarget 6: 34342-34357). The unique amino terminal sequences of the neuron-specific LNX1p70 and LNX1p62 variants are indicated in red text above the alignment (translation of p62 begins with the methionine in red font in the alignment). The alignment was prepared using Clustal Omega (https://www.ebi.ac.uk/Tools/msa/clustalo/) and displayed using MView (https://www.ebi.ac.uk/Tools/msa/mview/).

```
               1         .         .         .         .         :         .         .         . 80
Zebrafish LNX1  -----MPVCGATGVTPTAPPPDLCHICGQRHLQEENHEYTYKEDVDDDLMCHICLQPLIRPLDTPCGHTYCQECLTNFLL
Mouse LNX1p80   ------MNQPDLADDPDPSPEPLCIVCGQNHSPEENHFYTYTEDVDDDLICHICLQALLDPLDTPCGHTYCTLCLTNFLV
Zebrafish LNX2b MTES-----KTSSLPTGVTWARVCKECGQCHDGQDSHLFEYQDEVDDELVCHICLQPLLQPMDTPCGHTYCFQCLSNFLH
Zebrafish LNX2  MGSLGEGVDP--GMVSEALLEALCSECGQIHRTWENHLYNYRLEVDDDLVCHICLQPLVQPLDTPCGHTFCARCLRSFLQ
Mouse LNX2      MGTTSDEMVPVEQASSTSSLDPLCFECGQQHWARENHLYNYQGEVDDDLVCHICLQPLLQPLDTPCGHTFCHKCLRNFLQ
                                    {--*--*---*-ZnF-*--}         {*--*----- RING-*-*--*--*------


                          Mouse LNX1p70   MKALLLLVLPWLSPANYIDNVGNLHFLYSEL->
              81         .         :         .         .         .         .         :         . 160
Zebrafish LNX1  ESDFCPVDRTPLMLQKCRKSSLLVHKLLDKLMVSCPFAEHCTEVMPRGEMEGHIRCRCKGASHYGLSAERKRRSQEGDCT
Mouse LNX1p80   EKDFCPVDRKPVVLQHCKKSSILVNKLLNKLLVTCPFTEHCTEVLQRCDLQHHFQTSCKGASHYGLTKDRKRRSQDG-CP
Zebrafish LNX2b DQDFCPVDRQRIQLQQCRASSLLVRNLLDKLAVLCPFRSECELSMQRCELQPHLHNRCPAFKRLREEAERKKRPSWNE-L
Zebrafish LNX2  ERDFCPLDRAHLQLQVCRRSSILVHKLLDKLSVTCPLTPSCSLSMPRCDLEAHLKHRCPGTQSQRTKLERTQMEGSEDRV
Mouse LNX2      EKDFCPLDRKRLHFKLCKKSSILVHKLLDKLLVLCPFSPVCQDVMQRCDLEAHLKNRCPGASHRRVDLERRKTSQTQTQI
                ----*--*}                    {----*-----*---- ZnF---*----*--}


         Mouse LNX1p62->
             161         .         .         .         :         .         .         .         . 240
Zebrafish LNX1  DSTSELTLAA--------------LPGE-GCPSSAIALLSDEPGLVNPAYEPSVEDNSQSGSTTSLAARSGSRKNRNFDR
Mouse LNX1p80   DGCASLMATT--------------LSPE-VSAAATISLMTDEPGLDNPAYVSSVEDGEPVANS-SDSGRSNRTRARPFER
Zebrafish LNX2b KGSKSDGDLADAKSA-------------QTIGRTAQLSAPSEPGLVNPAFEEGEDDTPLR---CS---------------
Zebrafish LNX2  ------TATNPPKSPQTEIKEDTPSPPAGSNACNNVPTWTEEPGIDNPAFEESTEEDSVHGLECALPRV-----KRPLSN
Mouse LNX2      EGETGSTVIDPPGTLPPETDCSGTVPGERNLTPASLPVWTEEPGLDNPAFEESAAADSVQ--------------------
                                                             {NPAY/F}

             241         :         .         .         .         .         :         .         . 320
Zebrafish LNX1  TSVRSRSFRRLNRAFSVLRRTKSGTAVANDTTEERDNLRNANIPAEVFALPQLHHLIPDGEVTSIKITRADPCEPLAISI
Mouse LNX1p80   STMRSRSFKKINRALSALRRTKSGSVVANHVDQGRDNSENTTVPE---VFPRLFHLIPDGEITSIKINRADPSESLSIRL
Zebrafish LNX2b ---------------------------------------------------------LVAEATVVELFREDPGEDLGLRI
Zebrafish LNX2  ------------PCIHLLR-TGSSA------SSGWDFVETLPLSAE--EGCVKLPSLPEGEITTIEVHRTNPYSEMGISI
Mouse LNX2      ----------------------------------------------------QPLSLPEGEITTIEIHRSNPYIQLGISI
                                                                                        {-------
```

```
                  321                                                                              400
Zebrafish LNX1    VGGNETPLVRILIQDIYREGVIARDGRLLPGDMILKVNGIDISNVPHCYAVAALKQPCTLLRLTVLREQRHRYRSHHHSP
Mouse LNX1p80     VGGSETPLVHIIIQHIYRDGVIARDGRLLPGDIILKVNGMDISNVPHNYAVRLLRQPCQVLRLTVLREQKFRSRSNAHVP
Zebrafish LNX2b   VGGKDTPLGNIVIQEIVRDSLVARDGKLAPGDHILEVNDVSLASISHSRAIAVIRQPCSRLRLTVMQEKGFKPRPEHHTQ
Zebrafish LNX2    VGGNETPLINVVIQEVYRDGVIARDGRLLAGDQILQVNNVDISNVPHNFARSTLARPCATLQLTVLRERRCSARPPAATA
Mouse LNX2        VGGNETPLINIVIQEVYRDGVIARDGRLLAGDQILQVNNYDISNVSHNHARAVLSQPCSTLQLTVLRERRFGSRANSHAD
                  ------------------------ PDZ1----------------------------------------}

                  401                                                                              480
Zebrafish LNX1    TEPFPA----HTATIRDDSLHVVLVKRAPDEQLGIKLVRRPDEHGVFIFHLLEGGLAARDGRLRVDDRVLAINGHDLRYG
Mouse LNX1p80     DS----------YGPRDDSFHVILNKSSPEEQLGIKLVRRVDEPGVFIFNVLNGGVADRHGQLEENDRVLAINGHDLRFG
Zebrafish LNX2b   PSASPPTQSPSTNQNHGTVIQVTLVKHERSEALGIKLIRKSEEPGVFILDLLPGGLAAKDGKLRNNDKVLGINGQDLRHG
Zebrafish LNX2    SPKG-----------SPASIRITLHKRESSEQLGIKLVRRTDEAGVFILDLLEGGLAAKDGRLCSNDRVLAVNEHDLRHG
Mouse LNX2        GSAP-----------RDEVFQVLLHKRDSTEQLGIKLVRRTDEPGVFILDLLEGGLAAQDGRLNSNDRVLAINGHDLKHG
                                              {--------------- ---------- PDZ2--------------------

                  481                                                                              560
Zebrafish LNX1    APEHAALLIQASEDRVHFIVSRQTHIPAP----DILQEAPWSMEGPPPYS-----------PVDIEHTLLDSCQKPACYE
Mouse LNX1p80     SPESAAHLIQASERRVHLVVSRQVRQSSP----DIFQEAGWISNGQQSPG-----------PGERNT---ASKPAATCHE
Zebrafish LNX2b   TPESAAQIIQASEMRVNFVVMRLQDVSEEGGEGQSRGA--------------RRVPEPQYFRRHSEYMKEPPGGFSSQE
Zebrafish LNX2    TPELAAQIIQASGERVNLLISRSSKQTMAVHTGSTLTRDIWSHDHIPPLPSTATPSPVPSLHLARSSTQRDLSQCVNCKE
Mouse LNX2        TPELAAQIIQASGERVNLTIARPGKPQPSNGSRE--AGAHSSSNHAQ-----------PPSHSRPGSHKDLTRCVTCQE
                  ----- PDZ2--------------}

                  561                                                                              640
Zebrafish LNX1    KTVTLLKEPHDSLGMTVAGGMSSRGWDLPVYVTNVDPNGVVGQEGSIRKGDILLNVNGVDLTGVTRSEAVANLKNT--SS
Mouse LNX1p80     KVVSVWKDPSESLGMTVGGGASHREWDLPIYVISVEPGGVISRDGRIKTGDILLNVNGIELTEVSRTEAVAILKSA--PS
Zebrafish LNX2b   KTVTLKKEPRQSLGITIAGGRDCRS-RLPVYITSVQPVGCLHRDGTVKTGDVLLSINGIDLTHLTYNEAVTVLKTQTAQS
Zebrafish LNX2    KHITVKKEPHESLGMTVAGGRGSKSGELPIFVTSVQPHGCLSRDGRIKRGDVLLSINGQDLTYLSHSEAVGTLKSSATSC
Mouse LNX2        KHITVKKEPHESLGMTVAGGRGSKSGELPIFVTSVPPHGCLARDGRIKRGDVLLNINGIDLTNLSHSEAVAMLKASAASP
                           {----------------------------------PDZ3------------------------------
                  641                                                                              720
Zebrafish LNX1    PVVLQVLEMRPPNESSLDCMPPLHSPCALSPSSPGDVKLPPPNDDYAPLWVSWLQLPRHLYCCKDIVFRRSTSGSLGFSI
Mouse LNX1p80     SVVLKALEVKEQE-AQEDCSPAA----------LDSNHNVTPPGDWSPSWVMWLELPQYLCNCKDVILRRNTAGSLGFCI
Zebrafish LNX2b   TVTLRVIQTFADDEEQDGESGSRE----DMD---TMEGPKDDDINWVPLWTRWLGLPSCIHWCRDIMLMKTNSESWGFSI
Zebrafish LNX2    SVQLKALEVTMVEEPGLD----EE----L-----LPPHENDYDASWSPSWVLWLGLPSYLHSSHEIVLRRSHPGSWGFSI
Mouse LNX2        AVILKALEVQIAEEAAQA----TE----EQP---SAFSENEYDASWSPSWVMWLGLPSALHSCHDIVLRRSYLGSWGFSI
                  ----------}                                                                 {-------

                  721                                                                         795
Zebrafish LNX1    VGGQEELNCNQSFFIRSIVEGTPAYNDGRIRCGDILLEVNGKSTWGMTHTALVRLLKELRGRITLTIVSWPGSLL
Mouse LNX1p80     VGGYEEYSGNKPFFIKSIVEGTPAYNDGRIRCGDILLAVNGRSTSGMIHACLARMLKELKGRITLTIASWPGTFL
Zebrafish LNX2b   VGGYEESRGQQPFFIKTIVPGTPAYFDGRLKCGDEIVAVNGVTTVGMNNSSLIPMLKLQKNKVTLTVVSWPGSLV
Zebrafish LNX2    VGGYEENHSNQAFFIKTIVLGTPAYYDGRLKCGDMIVAVNGLSTAGMSHSALVPMLKEQRSRVALTVVSWPGSLI
Mouse LNX2        VGGYEENHTNQPFFIKTIVLGTPAYYDGRLKCGDMIVAVNGLSTVGMSHSALVPMLKEQRNKVTLTVICWPGSLV
                  ------------------------ PDZ4----------------------------------------}
```
